# Effect of Earthworm on Wound Healing: A Systematic Review and Meta-Analysis

**DOI:** 10.3389/fphar.2021.691742

**Published:** 2021-10-22

**Authors:** Dong Wang, Zhen Ruan, Rongchao Zhang, Xuejing Wang, Ruihui Wang, Zhishu Tang

**Affiliations:** ^1^ Medical Research and Experiment Center, Shaanxi University of Chinese Medicine, Xianyang, China; ^2^ Shaanxi University of Chinese Medicine, Xianyang, China

**Keywords:** earthworm extracts, wound healing, meta-analysis, systematic review, traditional Chinese medicine

## Abstract

**Background:** Earthworm, also called dilong (Chinese language), has been used as a traditional Chinese medicine for thousands of years. Recently, some scientists believe that earthworm extracts (EE) can promote wound healing. However, its effectiveness remains controversial. We conducted a meta-analysis to evaluate the effect of EE on wound healing based on the healing rate.

**Methods:** We comprehensively reviewed literature that mentioned EE for wound healing in the PubMed, Cochrane Library, Embase, Web of Science, China National Knowledge Infrastructure (CNKI), VIP database for Chinese Technical Periodicals, and WanFang database that have been published until January 2021. We computed weighted mean difference (WMD) for analysis with RevMan 5.3 software in animal and human models groups. Two researchers independently selected studies and evaluated the risk of bias with the Cochrane Collaboration tool. The quality of the evidence was assessed with the Cochrane risk of bias tool. This study is registered on PROSPERO (CRD42020168400).

**Results:** From 2,486 articles, we selected 16 studies for analysis. EE treatment was associated with improvements in wound healing performance based on wound healing rate (mouse model: weighted mean difference (WMD) = 3.55, 95% confidence interval (CI): 2.34–4.77, *p* < 0.00001; rat model: WMD = 17.29, 95% CI: 5.75–28.82, *p* = 0.003; rabbit model: WMD = 19.29, 95% CI: 9.95–28.64, *p* < 0.0001). Clinical studies also confirmed that EE could reduce healing time in hospital (WMD = −8.94, 95% CI: −17.75 to −0.14, *p* = 0.05).

**Conclusion:** The results of this meta-analysis demonstrated the efficacy of EE on wound healing process. As a corollary, EE can be a useful natural product for wound healing drug development.

**Systematic Review Registration** **:** https://www.crd.york.ac.uk/PROSPERO/display_record.php?RecordID=168400, identifier CRD42020168400.

## Introduction

The skin is the largest organ that protects the host from the external environment and maintains homeostasis (Richmond and Harris, 2014). Wounds provide entry points for exogenous materials and organisms into the body. Wound healing is a complex coordinated and intricate biological process that produces inflammation, proliferation, and remodeling phases (Campos et al., 2008). In clinics, physicians usually use antibiotics to treat injuries to prevent traumatic infections (Rowan et al., 2015). Although the wound healing process has been extensively studied, developing effective therapeutics for wound treatment remains difficult due to its complexity. Natural products are potential chemotherapeutic drugs for wounds (Kim et al., 2019). In China, earthworms (*Lumbricus* spp.), also called dilong, are used to treat various ailments, including burns, arthritis, itching, and inflammation. Earthworm extracts (EE) are important in the wound healing process (Matausic-Pisl et al., 2010; Bigham-Sadegh et al., 2016).

Establishing the acceleration effect of EE on wound closure by wound repair can be the first step toward drug design and future wound healing therapeutics. In this review, we analyzed the studies, including animal models and clinical trials, on the effect of EE on wound healing in comparison with conventional treatments, such as Jingwanhong (a traditional Chinese medicine) and normal saline. The data of wound healing rate were extracted and studied using the RevMan5.3 (Review Manager software, version 5.3, Copenhagen: The Nordic Cochrane Centre, The Cochrane Collaboration, 2014) and Stata 15.1 (Stata statistical software, version 15.1, Stata Corporation, College Station, Texas). This study aimed to determine the accelerated wound contraction potentials of EE on wound repair through a meta-analysis and systematic review. The outcome of this research is particularly important for the consideration of treatments for injury and for the development of new drugs from earthworm extracts.

## Materials and Methods

The protocol was registered on the international prospective register of systematic reviews PROSPERO (CRD42020168400) and can be accessed at https://www.crd.york.ac.uk/PROSPERO/display_record.php?RecordID=168400. The PRISMA 2020 (Preferred Reporting Items for Systematic Reviews and Meta-Analyses) and AMSTAR 2 for assessing the methodological quality of systematic reviews were used as guidelines in identifying and selecting relevant studies (Page et al., 2021).

### Search Strategy

The electronic databases (Embase, Web of Science, the Cochrane Library, PubMed, China National Knowledge Infrastructure (CNKI), VIP Database for Chinese Technical Periodicals (VIP), and WanFang) were searched by two independent researchers. All the databases were searched from their respective inceptions to January 2021. The following terms were used for search: “wound healing” OR “regeneration” OR “injury repair” OR “wound repair” OR “wound epithelialization” AND “Oligochaeta” OR “Dilong” OR “Earthworm” OR “Lumbricus terrestris” OR “*Eisenia* worm” OR “*Eisenia foetida*”. Study selection was restricted to the English and Chinese languages. Correspondence authors were contacted for unpublished studies when possible; however, no unpublished works were available because of two reasons: 1) No unpublished results satisfied the inclusion criteria. 2) The authors preferred not to disclose their work until it was submitted for publication. A detailed search strategy is presented in Supplementary Material S1.

### Study Selection

The criteria for the selection of studies for our meta-analysis were as follows: 1) All studies should be designed with randomized controlled trials (RCT), and original data were available. 2) EE was used to cure wound healing. 3) Studies should have cognitive performance outcomes measured by wound contraction (animal models) or healing time (clinic trials). 4) Oral medication or normal saline was used for comparison. The exclusion criteria were 1) review or meta-analysis articles; 2) uncorrelated to the effect of EE on wound healing articles; 3) reporting duplicated data articles; 4) wound healing rate or healing days not reported in articles.

### Data Extraction

Two investigators (Dong Wang and Zhen Ruan) independently reviewed and selected the studies based on the inclusion criteria. First author, publication year, species, number of experiments and controls, countries, and treatment methods for each group were identified and extracted from the method sections of each study. For animal model studies, wound contraction percentages (%) were extracted on the 10th day after injury. Alternatively, the nearest days were used instead, if the 10th day data were unpublished. For the clinical group, the healing time (days) and the rate of the growth of epidermis (%) were extracted for further analysis. Some simple arithmetic conversions were applied if the original data were not presented as wound healing rate or the format was not “mean ± standard deviation.”

### Methodological Quality and Assessment of Studies

The risk of bias for all studies was assessed using the modified tool for “risk of bias” from the Cochrane Handbook for Systematic Reviews of Interventions, which was obtained from the RevMan5.3. Several aspects of included studies assessed by the two researchers (Dong Wang and Zhen Ruan) were random sequence generation; allocation concealment; blinding of participants and personnel; blinding of outcome assessment; incomplete outcome data; selective reporting; and other biases. A level of “high,” “low,” or “unclear” was given for each item. Any disagreement was discussed with a third investigator (Xuejing Wang). Because of some different aspects of animal studies and RCT, the SYRCLE’s risk of bias tool based on the Cochrane Collaboration risk of bias tool was also applied to animal studies (Hooijmans et al., 2014).

### Data Analysis

RevMan 5.3 was used to analyze the data and generate forest and funnel plots. The pooled estimate was reported as weighted mean differences (WMDs) with 95% confidence intervals (CIs) for continuous outcomes. Either fixed-effect or random effect model was used to pool the effect sizes: if I^2^ < 50% and *p* ≥ 0.1, the pooled outcomes were calculated by the fixed-effects model; otherwise, a random-effects model was applied. Stata15.1 software was used to check publication bias. Publication bias was assessed using the funnel plots and Egger and Begg’s tests. We only checked publication bias in the mice and rat groups. The publication bias was not checked in other groups because of the number of enrolled studies; that is, rabbit and human groups only included three or less studies. Heterogeneity tests, including the Q and I^2^ statistics, were calculated; 25, 50, and 75% I^2^ scores were considered low, moderate, and high heterogeneities, respectively. Groups were categorized according to patients or animal models.

## Results

### Study Selection and Characteristics

**FIGURE 1:**
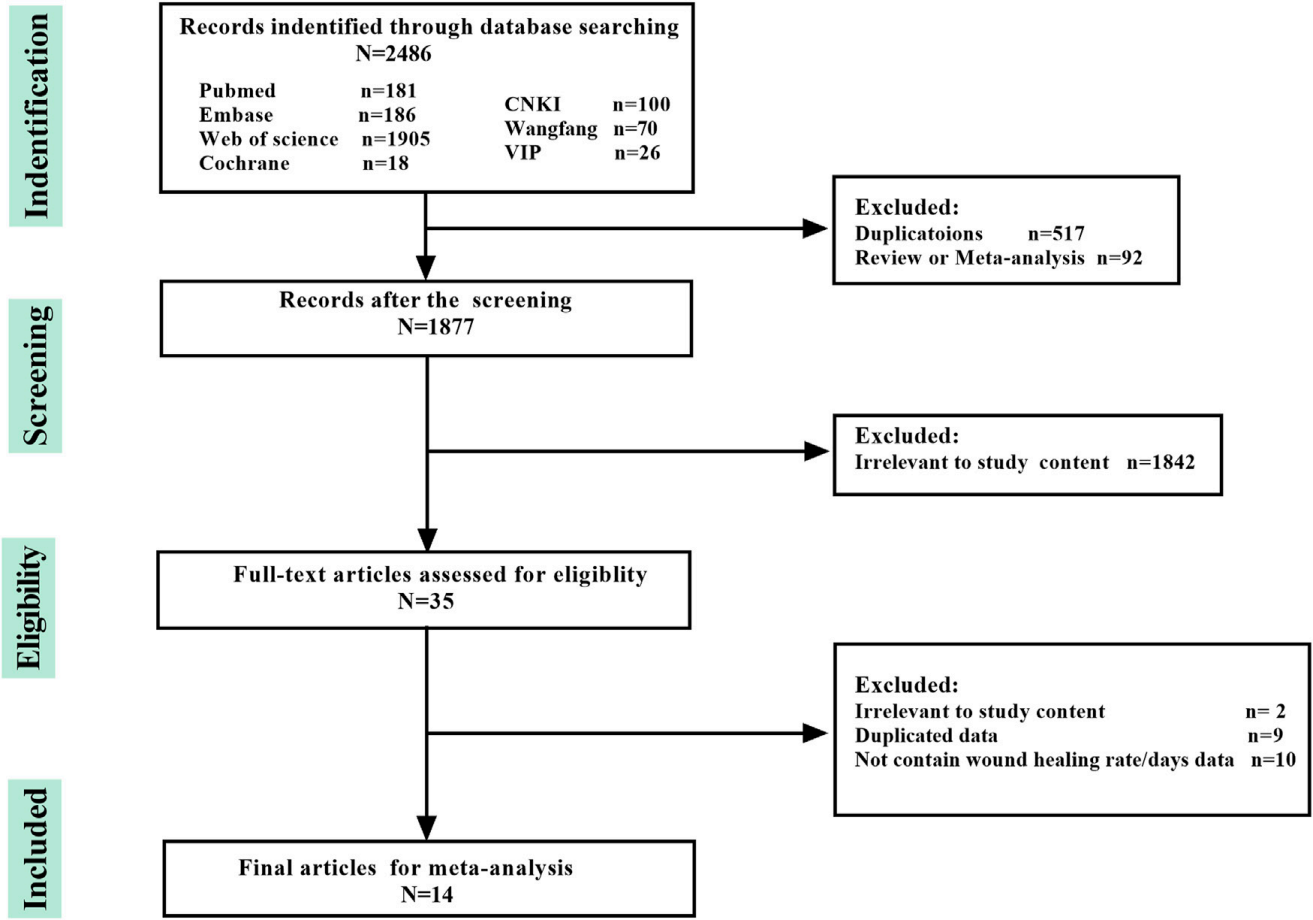
Flowchart of study search.

Our search obtained 2,486 articles: 181, 18, 186, 1905, 100, 26, and 70 articles from PubMed, Cochrane database, Embase, Web of Science, CNKI, VIP database, and WanFang database, respectively. Finally, 14 articles (including 16 studies), with eleven and three articles in the Chinese and English languages, respectively, matched the inclusion criteria. The protocols of the literature search and study selection are summarized in Figure 1. Among the 2,486 articles, 517 repeated articles and 92 reviews/meta-analyses were removed. After screening the titles and abstracts, 1,842 articles unrelated to EE and wound healing were excluded. Finally, 35 articles were obtained for further research. A total of 21 articles were excluded (Supplementary Material S5), as follows: two articles uncorrelated to the effect of EE on wound healing; nine articles reported duplicate data; and 10 articles did not report wound healing rate (in animal model studies) or healing days (in clinical studies). The earliest included study was published in 1999, and twelve studies were presented after 2010. These articles were distributed among four countries, namely, India, Iran, United States, and China. The article of Deng et al. (2018) was written by authors from India, United States, and China. Among the 16 studies, seven, four, and three studies used mouse, rat, and rabbit models, respectively, and two studies were from clinical trials. The article of Li D. et al. (2000) provided two studies: one was from rabbit model experiments and the other was from clinical trials. Li F. et al. (2020) also provided two studies: one intervention group applied earthworm syrup and another one applied earthworm syrup nanosilver hydrogel patch. A total of seven mouse model studies contained Kunming mouse from the Institute of Cancer Research mouse and Balb/c mouse. Female Wistar and male Sprague-Dawley rats were used in the rat model studies. A total of 30 New Zealand and 16 Japanese white rabbits were used in the three rabbit model studies. In clinical studies, a total of 205 patients were enrolled in this meta-analysis with sample sizes ranging from 34 to 71. All participants were randomly assigned to the intervention group or control group. In this meta-analysis, the intervention methods of studies were EE, and the EE’s form and adjuvant were ignored. For example, in the study of Wang et al. (2020), EE was carried into nanomaterials, but we also consider that the intervention method is EE in analysis. Normal saline was commonly used as control (31.25%), and Jingwanhong (main compositions: *Angelica sinensis*, *Angelica dahurica*, *Boswellia carterii* Birdw., *Arnebia guttata* Bunge, and *Commiphora myrrha* Engl.) was used in three studies (18.75%). Gels, ethacridine lactate, and povidone iodine were used twice separately. Tris-HCl and panthenol-D were used once separately in mouse and rat models (Table 1).

**TABLE 1:** Characteristics of studies used in meta-analysis.

Studies	Species	Age/Weight	Sample size	Intervention	Control	Outcome measurement	Sampling time	Country
Li F. et al. (2020)	KM mouse (B)	28–36 G	Ne = 15; Nc = 15	EE + syrup + nanosilver	Jingwanhong	WHR (mean ± SD)	11 days	China
Li F. et al. (2020)	KM mouse (B)	28–36 G	Ne = 15; Nc = 15	EE + syrup (1:0.8)	Normal saline	WHR (mean ± SD)	11 days	China
Zhou (2015)	KM mouse (B)	25–35 G	Ne = 20; Nc = 20	EE	Jingwanhong	WHR (mean ± SD)	11 days	China
Deng et al. (2018)	KM mouse (B)	6–8 W	Ne = 20; Nc = 20	EE	Jingwanhong	WHR (mean ± SD)	11 days	China, Iran, United States
Hu et al. (2012)	ICR mouse (M)	24–30 G	Ne = 6; Nc = 6	EE	0.01 M PH7.2 Tris-HCl	WHR (mean ± SD)	10 days	China
Wang et al. (2020)	Balb/c mouse (M)	5 W	Ne = 30; Nc = 30	EE + nanomaterials	Normal saline	WHR (mean ± SD)	9 days	China
Wang et al. (2015)	ICR mouse (M)	24–30 G	Ne = 6; Nc = 6	EE	Normal saline	WHR (mean ± SD)	10 days	China
Goodarzi et al. (2016)	Wistar rats (F)	Adults	Ne = 6; Nc = 6	EE	Panthenol-D	WHR (mean ± SD)	9 days	Iran
Zhang et al. (2019)	SD rat (M)	180–220 G	Ne = 24; Nc = 24	EE	Normal saline	WHR (mean ± SD)	9 days	China
Zhou et al. (2010)	Wistar rats	200–250 G	Ne = 28; Nc = 28	EE	Gels	WHR (mean ± SD)	12 days	China
Xi et al. (2011)	Wistar rats	200–250 G	Ne = 30; Nc = 30	EE	Gels	WHR (mean ± SD)	12 days	China
Amarpal et al. (2015)	NZW rabbit (B)	10–12 M/1.5–2.0 KG	Ne = 16; Nc = 16	EE	Povidone iodine	WAS(mean ± SE)	14 days	India
Li D. et al. (2000)	JW rabbit (B)	3.0–3.5 KG	Ne = 30; Nc = 30	EE	Ethacridine lactate	WAS(mean ± SD)	9 days	China
Zhang et al. (1999)	NZW rabbit (B)	2.37 ± 0.06 KG	Ne = 3; Nc = 3	EE	Normal saline	WHR (mean ± SD)	11 days	China
Bo and Pan (2012)	Patients (B)	24.8–49.3 Y	Ne = 35; Nc = 34	EE	Silvadene + povidone iodine	HD (mean ± SD)	--/3rd days	China
						/RGE		
Li D. et al. (2000)	Patients (B)	22–63 Y	Ne = 71; Nc = 65	EE	Ethacridine lactate	HD (mean ± SD)	--/7 days	China
						/RGE		

KM, Kunming; ICR, Institute of Cancer Research; SD, Sprague-Dawley; NZW, New Zealand White; JW, Japanese White; B, both sexes; M, male; F, female; W, weeks; M, months; Y, years; G, grams; KG, kilograms; Ne, number of experiments; Nc, number of controls; EE, extracts from earthworm; WHR, wound healing rate; WAS, wound areas surface; HD, healing days; RGE, rate of growth of epidermis.

### Methodologies for the Bias of Selected Studies

**FIGURE 2:**
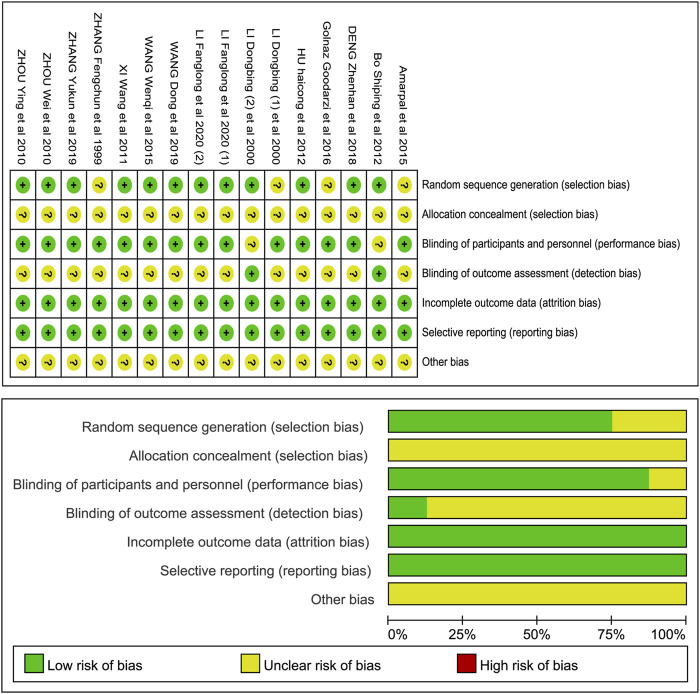
Risk of bias summary: review authors’ judgments about each risk of bias item for each included study.

The quality of enrolled studies is presented in Figure 2 and the quality of animal studies is shown in Table 2. Twelve studies clearly reported that random sequence generation was used in the research except in four studies. The risk bias of allocation concealment could not be assessed due to the lack of information from these studies. Two studies from clinical trials showed unclear risks in performances bias because the articles had insufficient information. Animals cannot understand the experiments even if the researcher explained the protocols to them in person, so the risk of performance bias is very low in animal models. The outcomes of animal studies were measured by the researchers, but we were unable to obtain enough information to assess the risk bias of blind outcome assessment. The data on the healing days of clinical trials were collected by the researchers and patients, and the risk was reduced. Incomplete outcome data were not reported, and all studies conducted selective reporting with a low risk of bias. No other types of bias were clearly indicated in general.

**TABLE 2:** SYRCLE’s tool for assessing the risk of bias in animal studies.

Studies	^a^Was the allocation sequence adequately generated and applied?	Were the groups similar at baseline or were they adjusted for confounders in the analysis?	^a^Was the allocation adequately concealed?	Were the animals randomly housed during the experiment?	Were the caregivers and/or investigators blinded from knowledge about intervention each animal received during the experiment?	Were animals selected at random for outcome assessment?	Was the outcome assessor-blinded?	^a^Were incomplete outcome data adequately addressed?	^a^Were reports of the study free of selective outcome reporting?	^a^Was the study apparently free of other problems that could result in a high risk of bias?
Li F. et al. (2020)	Low	Low	Unclear	Low	Unclear	Unclear	Low	Low	Low	Unclear
Li F. et al. (2020)	Low	Low	Unclear	Low	Unclear	Unclear	Low	Low	Low	Unclear
Zhou (2015)	Low	Low	Unclear	Unclear	Unclear	Unclear	Low	Low	Low	Unclear
Deng et al. (2018)	Low	Low	Unclear	Low	Unclear	Unclear	Low	Low	Low	Unclear
Hu et al. (2012)	Low	Low	Unclear	Unclear	Unclear	Unclear	Low	Low	Low	Unclear
Wang et al. (2020)	Low	Low	Unclear	Unclear	Unclear	Unclear	Low	Low	Low	Unclear
Wang et al. (2015)	Low	Low	Unclear	Unclear	Unclear	Unclear	Low	Low	Low	Unclear
Goodarzi et al. (2016)	Unclear	Low	Unclear	Low	Unclear	Unclear	Low	Low	Low	Unclear
Zhang et al. (2019)	Low	Low	Unclear	Unclear	Unclear	Unclear	Low	Low	Low	Unclear
Zhou et al. (2010)	Low	Low	Unclear	Unclear	Unclear	Unclear	Low	Low	Low	Unclear
Xi et al. (2011)	Low	Low	Unclear	Unclear	Unclear	Unclear	Low	Low	Low	Unclear
Amarpal et al. (2015)	Unclear	Low	Unclear	Unclear	Unclear	Unclear	Low	Low	Low	Unclear
Li D. et al. (2000)	Unclear	Low	Unclear	Unclear	Unclear	Unclear	Low	Low	Low	Unclear
Zhang et al. (1999)	Unclear	Low	Unclear	Unclear	Unclear	Unclear	Low	Low	Low	Unclear

a Items in agreement with the items in the Cochrane Risk of Bias tool.

### Effects of EE in Animal Model

**FIGURE 3:**
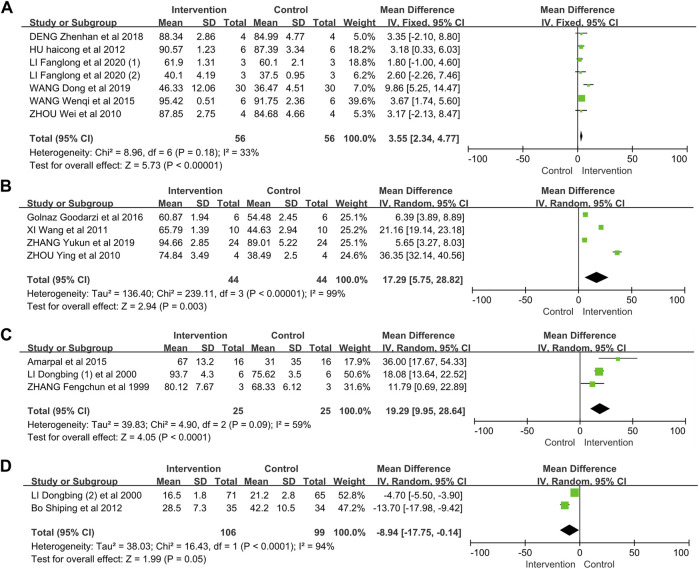
Meta-analysis on earthworm extract for wound healing. **(A)** Wound healing rate on mouse model; **(B)** wound healing rate on rat model; **(C)** wound healing rate on rabbit model; **(D)** healing time on clinic trials.

In summary, the selected 14 animal model studies were divided into three groups for meta-analysis. Half of the 125 animals were cured in the EE group, and others were treated in the control group. In the animal model studies, EE treatment was associated with increased wound healing rate (mouse model: WMD = 3.55, 95% CI: 2.34–4.77; rat model: WMD = 17.29, 95% CI: 5.75–28.82; rabbit model: WMD = 19.29, 95% CI: 9.95–28.64). The heterogeneity in the pooled results was I^2^ = 33% (*p* = 0.18), I^2^ = 99% (*p* < 0.00001), and I^2^ = 59% (*p* = 0.09) in the mouse, rat, and rabbit models, respectively (Figure 3). The test for overall effect (Z) was 5.73 (*p* < 0.00001), 2.94 (*p* = 0.003), and 4.05 (*p* < 0.0001) in the mouse, rat, and rabbit model groups, respectively.

### Effects of EE on Clinical Trials

In this meta-analysis group, we included 205 patients in two studies (Figure 3D). A total of 106 patients (male = 49; female = 57) and 99 patients (male = 49; female = 50) were cured in the EE and drug groups, respectively. The results of meta-analysis confirmed that EE could reduce healing time (WMD = −8.94, 95% CI: −17.75–−0.14), with significant heterogeneity in the pooled results (I^2^ = 94%, *p* < 0.0001). The test for overall effect (Z) was 1.99 (*p* = 0.05).

### Publication Bias

**FIGURE 4:**
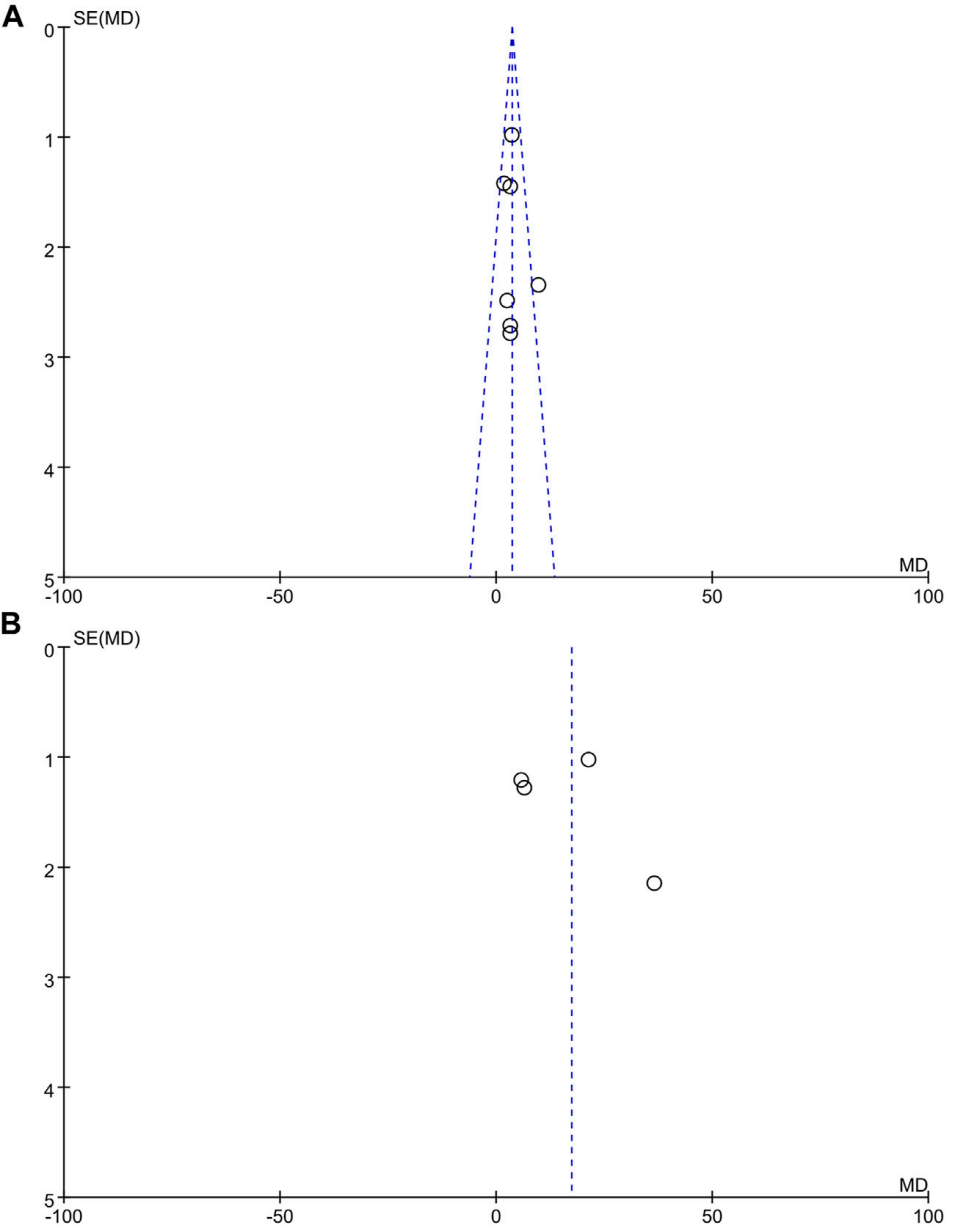
The funnel plot of earthworm extracts for wound healing rate. **(A)** Mouse model; **(B)** rat model.

Publication bias was only checked in the mouse and rat model groups because few articles were available in the other groups. We applied funnel plots, and Egger and Begg’s tests for outcomes to assess the potential publication bias of the included studies. With these studies, no evidence of extreme publication bias was observed (mouse model: Egger’s test: *p* = 0.593 and Begg’s test: *p* = 0.764; rat model: Egger’s test: *p* = 0.596 and Begg’s test: *p* = 0.734). The funnel plots are shown in Figure 4.

## Discussion

**FIGURE 5:**
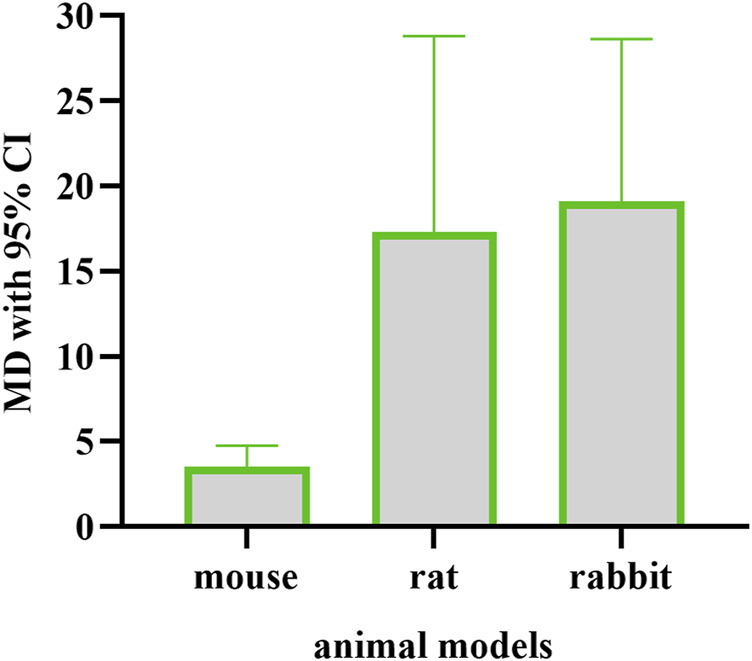
Mean differences in animal models.

In this systematic review and meta-analysis, eleven and three Chinese and English language articles, respectively, were enrolled after critical selection. A total of 16 studies from articles were divided into animal and clinical groups. The animal groups contained mouse, rat, and rabbit models. The intervention methods used EE to cure the wound and compared the control methods with saline normal or conventional drug. The meta-analysis results indicated that EE performed better than drug or normal saline in terms of wound healing rate (Figure 3). The rat and rabbit model group showed high WMD, indicating that the effect of EE on rabbit and rat models was better than that on mouse model (Figure 5). In the mouse model, when the study of Wang Dong et al. (2019) (Wang et al., 2020) was eliminated, no significant heterogeneity existed (I^2^ = 0%, *p* = 0.94). This finding may be due to the presence of nanomaterial, which was used in the study as a drug release carrier. The main effective component is EE, but the nanomaterial can protect the extract, thereby improving stability and increasing potency. The extract was applied at days 1 and 7 after the injury, and the extract was used once daily, so the test for overall effect was increased to Z = 4.79 (*p* < 0.00001) when this study was removed. The heterogeneity in the rat model showed significance with the I^2^ = 99% (*p* < 0.00001). This result may be due to the fact that only four studies were included in this group. However, there was no directional change in the overall results after included studies were systematically omitted in turn, which indicated that these results are credible. In the rabbit model study, if the study of Amarpal et al. (2015) was excluded, then the heterogeneity was reduced to I^2^ = 6% (*p* = 0.30). Thus, this study was the main source of heterogeneity in the analysis. This result may be due to the fact that the sampling time (on the 14th day after injury) in this study was later than the sampling time (9th and 11th days) in other studies. The healing rate was slower with increasing number of days. So, the overall effect increased (Z = 8.19, *p* < 0.00001), and the WMD (95% CI) was reduced to 17.21 (13.09–21.33) when the study of Amarpal et al. (2015) was moved. The strains and weight of rabbits in these studies may also cause heterogeneity. The studies from clinical trials had high heterogeneity (I^2^ = 94%, *p* < 0.0001), because the types of injury differed. In the study of Bo Shiping et al. (Zhou, 2015), the injuries of patients included 35 burn and scald wounds (50.72%), 10 operation wounds (14.49%), 10 bedsore wounds (14.49%), and chronic ulcer wounds (20.29%). In the study of Li Dongbing et al. (Deng et al., 2018), the wounds are surgical injuries. The gap of the publishing years of the two studies was more than 10 years, during which the clinical environment and technology improved. Furthermore, the control drugs also differed. These discussed factors can increase the heterogeneity in this group. Although with high heterogeneity, the pooled results indicated that the EE could accelerate wound healing in clinic.

**FIGURE 6:**
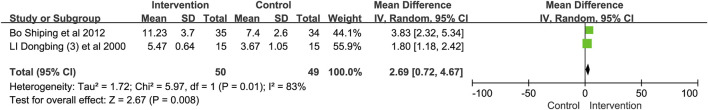
Meta-analysis on earthworm extract for rate of growth of epidermis.

The articles of Li Dongbing et al. (Goodarzi et al., 2016) and Bo Shiping et al. (Bo and Pan, 2012) reported the rate of the growth of epidermis, and they also investigated the healing rate. The pooled results (Figure 6) present that the WMD (95% CI) was 2.69 (0.72, 4.67), the I^2^ was 83% (*p* = 0.01), and the effect size was Z = 2.67 (*p* = 0.008). These results indicated that EE can accelerate cell proliferation in the wound healing process.

In modern society, the number of patients with various injuries caused by traffic accidents and accidental events (scald, mechanical injury, etc.) is increasing rapidly. In addition to traumatic injury, millions of surgical wounds are created annually in the course of routine medical care in the United States and Europe (Eming et al., 2014). Wound healing is an important physiological process to maintain the integrity of skin after trauma. At present, the main method to treat the wound is to inhibit the growth of microorganisms with antibiotics, but it has some side effects such as mild skin rash and upset stomach (Cunha, 2001). Therefore, postwound repair has always been a hot research topic in the medical field. In recent years, it has been found that dilong as a traditional Chinese medicine has a unique effect on wound healing. In the Qing Dynasty, it was clearly pointed out that the dilong could “cure bruise and pock” in the book “Hui Yue Yi Jing.” The results of our meta-analysis also showed that EE can promote wound healing, which lays a foundation for future research and clinical application of EE in wound healing.

In this investigation, we also found that some researchers characterized other indexes that are not enrolled in the meta-analysis to indicate the effect of EE on wound healing (Table 3). The indexes are related to cytokines, amino acids, and cell proliferation rate. Mouse, rat, rabbit, and cells are used to test the effect of earthworm in these studies. Wei et al. (2009) also reported that EE may enhance sciatic nerve regeneration and function recovery after injury. Furthermore, Bigham-Sadegh et al. (2016) found that EE (G-90) can reduce inflammation and increase the maturity of fibrocytes and the aggregation of collagen fibers in tendons after tendon operation in rabbit. The results suggested the clinical potential of EE in the treatment of peripheral nerve injury in humans.

**TABLE 3:** Characteristics of earthworm extract for wound healing.

Studies	Characteristics	Results		Materials	Sampling time
		Intervention	Control		
Grdiša et al. (2004)	EGF (ng/ml)	55.2 ± 0.28	9.2 ± 0.28	NIH mouse (M)	24 h
	FGF (ng/ml)	22.5 ± 0.23	7.2 ± 0.19		
Zhang et al. (2006)	OCSS (mmHg)	4.77 ± 0.92	3.12 ± 1.26	Wistar rats	24 h
	SDH (OD)	0.62 ± 0.1	0.34 ± 0.09		
	FISB (OD)	26.9 + −0.6	29.8 + −0.9		
Chen et al. (2010)	LRNC	0.51 ± 0.03	0.42 ± 0.02	PC12 cells	72 h
	Synapsin I	175 ± 25%	100%		
	GAP-43	164 ± 70%	100%		
Hou (2013)	TNF-a (pg/ml)	257 ± 50	152 ± 30	Wistar rats	7 days
	IL-10 (pg/ml)	1,000 ± 150	625 ± 100		
	Malondialdehyde (mg protein/ml)	0.95 ± 0.1	0.73 ± 0.09		
	Hydroxyproline (ug/ml)	2.22 ± 0.15	1.62 ± 0.15		
Hu et al (2013)	GE (days)	16.12 ± 1.8	18.36 ± 2.23	JWR	--
Yang et al. (2017)	CPR	1.54 ± 0.05%	1%	NIH 3T3 cells	24 h
	CPR	1.85 ± 0.05%	1%	HaCaT cells	
Deng et al. (2018)	Platelet (10^9^/L)	504 ± 19.33	432 ± 6.35	KM mouse (B)	7 days
	WBC(10^9^/L)	13.07 ± 0.18	10.40 ± 0.59		
	Gran (10^9^/L)	325 ± 20.38	62 ± 9.31		
Zhang et al. (2019)	Total protein (g/L)	0.36 ± 0.15	0.17 ± 0.07	SD rat	7 days
	IL-6 (ng/L)	32.22 ± 5.32	38.29 ± 7.24		
	Hydroxyproline (ug/mg)	5.85 ± 1.41	4.07 ± 0.5		

EGF, epidermal growth factor; FGF, fibroblast growth factor; OCSS, oxygen consumption of scalded skin; SDH, succinate dehydrogenase; FISB, fluorescence intensity of Schiff’s base; LRNC, length ratio between neurite and cell body; GE, growth of epidermis; CPR, cell proliferation rate; KM, Kunming; SD, Sprague-Dawley.

Several limitations to the present study should be considered. First, in this investigation, only several articles are enrolled for meta-analysis. We included fewer than 10 studies in each group, which may influence the results. Since fewer than or equal to three studies were included in the rabbit and human groups, we only assessed publication bias in the mouse and rat model groups. Second, due to the unclear chemical compositions of the EE and the lack of unified treatment regimens, this may be one of the reasons for the high heterogeneity of the results. Third, this study involves fewer countries; most of the studies are from China. It also shows that there are few studies on the treatment of wound with EE in the world. With additional studies published, a larger sample study will further confirm its efficacy in the future. With additional studies published in the future, and clear results can be provided.

## Conclusion

Infection and inflammation are the common responses to injury, and antibiotics are usually used for treatment. However, the abuse of antibiotics decreases pharmacological efficacy and contraction of drugs. Our research shows a potential to discover new drugs from EE. Even with only 14 included articles, our meta-analysis further confirmed the effect of EE on wound healing process. In this review, we investigated 2,486 articles from seven databases and obtained 16 studies that met the inclusion criteria after critical selection. Then, the data from these studies were carefully analyzed, and we found that EE can reduce healing time and increase wound healing rate. The extracts can accelerate cell proliferation and multiplication after the injury according to biochemical and histomorphological experiments. Research also confirmed that the extracts have clinical potential for wound healing in clinic. Finding the main effect ingredients and creating a stable formation of EE should be the next step in this field.

## Data Availability

The original contributions presented in the study are included in the article/Supplementary Material; further inquiries can be directed to the corresponding author.

## Supplementary Material

Supplementary Material S1 Search strategy.

Supplementary Material S2 PRISMA 2020 Checklist.

Supplementary Material S3 AMSTAR 2 Checklist.

Supplementary Material S4 Original data.

Supplementary Material S5 Not included articles.
