# Supplementary material for: Effect of Earthworm on Wound Healing: A Systematic Review and Meta-Analysis
Source: Front Pharmacol. 2021 Oct 22;12:691742. doi: 10.3389/fphar.2021.691742 (PMC8568887; doi:10.3389/fphar.2021.691742)
Supplement: Supplementary file 1 [file DataSheet4.DOCX]

**S-Table1. Data of wound contraction percentages extracted from included studies in mouse model.**

| Study(mouse) | intervention | | total | control | | total |
| --- | --- | --- | --- | --- | --- | --- |
|  | mean | SD |  | mean | SD |  |
| DENG Zhenhan et al 2018 | 88.34 | 2.86 | 4 | 84.99 | 4.77 | 4 |
| HU haicong et al 2012 | 90.57 | 1.23 | 6 | 87.39 | 3.34 | 6 |
| LI Fanglong et al 2020 (1) | 61.9 | 1.31 | 3 | 60.1 | 2.1 | 3 |
| LI Fanglong et al 2020 (2) | 40.1 | 4.19 | 3 | 37.5 | 0.95 | 3 |
| WANG Dong et al 2019 | 46.33 | 12.06 | 30 | 36.47 | 4.51 | 30 |
| WANG Wenqi et al 2015 | 95.42 | 0.51 | 6 | 91.75 | 2.36 | 6 |
| ZHOU Wei et al 2010 | 87.85 | 2.75 | 4 | 84.68 | 4.66 | 4 |

**S-Table2. Data of wound contraction percentages extracted from included studies in rat model.**

| Study (rat) | intervention | | total | control | | total |
| --- | --- | --- | --- | --- | --- | --- |
|  | mean | SD |  | mean | SD |  |
| Golnaz Goodarzi et al 2016 | 60.87 | 1.94 | 6 | 54.48 | 2.45 | 6 |
| XI Wang et al 2011 | 65.79 | 1.39 | 10 | 44.63 | 2.94 | 10 |
| ZHANG Yukun et al 2019 | 94.66 | 2.85 | 24 | 89.01 | 5.22 | 24 |
| ZHOU Ying et al 2010 | 74.84 | 3.49 | 4 | 38.49 | 2.5 | 4 |

**S-Table3. Data of wound contraction percentages extracted from included studies in rabbit model.**

| Study(Rabbit) | intervention | | total | control | | total |
| --- | --- | --- | --- | --- | --- | --- |
|  | mean | SD |  | mean | SD |  |
| ZHANG Fengchun et al 1999 | 80.12 | 7.67 | 3 | 68.33 | 6.12 | 3 |
| *****LI Dongbing (1) et al 2000 | 93.7 | 4.3 | 6 | 75.62 | 3.5 | 6 |
| Amarpal et al 2015 | 67 | 13.2 | 16 | 31 | 35 | 16 |

**S-Table4. Data of healing time extracted from included studies in human model.**

| Study(human) | intervention | | total | control | | total |
| --- | --- | --- | --- | --- | --- | --- |
|  | mean | SD |  | mean | SD |  |
| Bo Shiping et al 2012 | 28.5 | 7.3 | 35 | 42.2 | 10.5 | 34 |
| LI Dongbing (2) et al 2000 | 16.5 | 1.8 | 71 | 21.2 | 2.8 | 65 |

**S-Table5. Data of the rate of the growth of epidermis extracted from included studies in human model.**

| Study(the rate of the growth of epidermis) | intervention | | total | control | | total |
| --- | --- | --- | --- | --- | --- | --- |
|  | mean | SD |  | mean | SD |  |
| Bo Shiping et al 2012 | 11.23 | 3.7 | 35 | 7.4 | 2.6 | 34 |
| LI Dongbing (2) et al 2000 | 5.47 | 0.64 | 15 | 3.67 | 1.05 | 15 |

***The data has been converted by some simple arithmetic. The original has been presented below.**

| groups | Wound area (cm^2^, initial area: 2.543 cm^2^) | Sample number |
| --- | --- | --- |
| Intervention | 0.16±0.11 | 6 |
| Control | 0.62±0.09 | 6 |

wound contraction percentages= [(initial area- wound area)/ initial area]×100%.
