# Supplementary material for: Effect of Earthworm on Wound Healing: A Systematic Review and Meta-Analysis
Source: Front Pharmacol. 2021 Oct 22;12:691742. doi: 10.3389/fphar.2021.691742 (PMC8568887; doi:10.3389/fphar.2021.691742)
Supplement: Supplementary file 2 [file DataSheet5.DOCX]

**S-Table 6. The 21 excluded articles**

| **Studies** | **Title** | **Country** | **Reasons for excluded** |
| --- | --- | --- | --- |
| Chen Yunfeng et al. 1998[1] | Morphological observation of earthworm promoting wound healing in rabbit model | China | uncorrelated to effect of EE on wound healing |
| Cui Hong 2004 et al.[2] | Experimental study on the effect of earthworm tissue components on fibroblast proliferation | China | uncorrelated to effect of EE on wound healing |
| Yin Jianjian 2015 et al.[3] | The research of earthworm anti-damage of effective components on the mouse trauma | China | reported duplicate data |
| Hu Haicong et al. 2012[4] | Research on the effect of regeneration extract of Earthworm on wound healing in mice | China | reported duplicate data |
| Chen Yunfeng et al. 2000[5] | Experimental study on promoting wound healing of Earthworm | China | reported duplicate data |
| Li Dongbing et al. 2000[6] | Experimental and clinical study on the effect of Earthworm on postoperative wound healing of hemorrhoids | China | reported duplicate data |
| Li Dongbing et al. 1998[7] | Experimental and clinical study on the effect of Earthworm on postoperative wound healing of hemorrhoids | China | reported duplicate data |
| Li Dongbing et al. 1999[8] | Clinical study on promoting wound healing of hemorrhoid after operation with Dylongium component solution | China | reported duplicate data |
| Tang Huaqi et al. 2014[9] | Observation of HE staining in promoting wound healing of mice with extract of Earthworm | China | reported duplicate data |
| Zhou Ying et al. 2010 [10] | Study on the effect of earthworm protein on scald | China | reported duplicate data |
| Wang Dong et al. 2020 [11] | Making Fe-Ni earthworm protein complex and its application in experimental rat/mouse trauma experiments | China | reported duplicate data |
| Zhang Fengchun et al. 1998[12] | Experimental study on the effect of Earthworm on back wound contraction in white rabbits | China | not report wound healing rate or healing days |
| Xin Mei et al. 2004[13] | Effect of Earthworm extract on deep ⅱ degree scald wound healing in rats | China | not report wound healing rate or healing days |
| Yu Xingbin et al. 2014 [14] | Observation on Curative Effect of Earthworm Extract in Treating Diabetic Foot | China | not report wound healing rate or healing days |
| Duan Xiaojie et al. 2018[15] | Study on the screening of active components in promoting wound healing of Earthearthling | China | not report wound healing rate or healing days |
| Wei Baozhen et al. 2015 [16] | To observe the clinical effect of earthworm extract liquid in treatment of diabetic foot | China | not report wound healing rate or healing days |
| Xie Maiqiu et al. 1999[17] | Experience of treating 22 cases of bedsore with earthworm sugar solution | China | not report wound healing rate or healing days |
| Sun Haiyan et al. 1998[18] | Clinical observation on external application of fresh dilong in treating traumatic ulcer | China | not report wound healing rate or healing days |
| Matausic-Pisl M et al. 2010 [19] | Tissue extract from Eisenia foetida as a wound-healing agent | Croatia | not report wound healing rate or healing days |
| Li Deming et al. 2000[20] | Observation on the effect of Dilong ointment on postoperative wound healing of anorectal disease | China | not report wound healing rate or healing days |
| Duan Xiaojie et al. 2018[21] | Active fraction of DL3 and its would healing study | China | not report wound healing rate or healing days |

1. Chen YF. Morphological observation of earthworm promoting wound healing in rabbit model. Chin J Chin Mat Med 1998; 23(5):305-307 (in Chinese)

2. Cui H, Yu PL, Sun L, Zhang ZX, Zhang HY, Li WH. Experimental study on the effect of earthworm tissue components on fibroblast proliferation Journal of Capital Medical University 2004; 25(3):317-320 (in Chinese)

3. Yin JJ**.** The research of earthworm anti-damage of effective components on the mouse trauma *[M.S. thesis]*. Hunan Agricultural University; 2015. (in Chinese)

4. Hu HC, LI CF, Zhang JY, Zhang XJ, Sun YK**.** Research on the effect of regeneration extract of Earthworm on wound healing in mice. The Fifth Academic Exchange Meeting of Chinese Medicine Analysis Branch of China Association of Chinese Medicine, 2012. Shenyang, Liaoning, China. 6. (in Chinese)

5. Chen YF, Zhang FC, Su YZ. Experimental study on promoting wound healing of Earthworm. Chinese Journal of Plastic Surgery 2000; 16(3):183-184 (in Chinese)

6. Li DB, Wang P, Zeng YH, Zhao YR, Qian JR, Chen XL, Zhao YQ, Wu ZZ. Experimental and clinical study on the effect of earthworm on postoperative wound healing of hemorrhoids. Chin J Bases Clin General Surg 2000; 7(1):6-9 (in Chinese)

7. Li DB**.** Experimental and clinical study on the effect of Earthworm on postoperative wound healing of hemorrhoids *[M.S. thesis]*. Beijing University of Chinese Medicine; 1998. (in Chinese)

8. Li DB. Clinical study on promoting wound healing of hemorrhoid after operation with Dylongium component solution. Chin J Coloproctol 1999; 19(11):3-5 (in Chinese)

9. Tang HQ, Hu HC, Wang WQ, Zhang ZQ, Li DD, Sun YK**.** Observation of HE staining in promoting wound healing of mice with extract of Earthworm. The 7th Academic Exchange Meeting on TCM analysis of China Association of Traditional Chinese Medicine, 2014. Guangzhou, Guangdong, China. 4. (in Chinese)

10. Zhou Y**.** Study on the effect of earthworm protein on scald *[M.S. Thesis]*. Shandong University; 2010. (in Chinese)

11. Wang D, Wang XJ, H WR, Yang H, Wang XY, Tang ZS. Making Fe-Ni earthworm protein complex and its application in experimental rat/mouse trauma experiments. China Journal of Traditional Chinese Medicine and Pharmacy 2020; 35(4):2027-2030 (in Chinese)

12. Zhang FC, Chen YF, Su YZ, Hu QF. Experimental study on the effect of Earthworm on back wound contraction in white rabbits. Chin J Chin Mat Med 1998; 23(9):49-50 (in Chinese)

13. Xin M, Li X, Ye F, Liu Y, Qiu G. Effect of Earthworm extract on deep ⅱ degree scald wound healing in rats. Journal of Chinese Medicinal Materials 2004; 27(10):744-747 (in Chinese)

14. Yu XB, C ZX, Xie ZN. Observation on Curative Effect of Earthworm Extract in Treating Diabetic Foot. World Chinese Medicine 2014; (2):196-198 (in Chinese)

15. Duan XJ, Yang YW, Geng D, Luo SL, F WX, Ding YT, Zheng ZH, Zhao RY, Sun YK. Study on the screening of active components in promoting wound healing of Earthearthling. Acta Chinese Medicine and Pharmacology 2018; 46(1):5-7 (in Chinese)

16. Wei BZ. To observe the clinical effect of earthworm extract liquid in treatment of diabetic foot. Diabetes New World 2015; (12):50-51 (in Chinese)

17. Xie MQ. Experience of treating 22 cases of bedsore with earthworm sugar solution. Journal of Hunan Normal University (Medical Science) 1999; (3):16-17 (in Chinese)

18. Sun HY, Jiang XM. Clinical observation on external application of fresh dilong in treating traumatic ulcer. Hebei Med 1998; (12):50 (in Chinese)

19. Matausic-Pisl M, Cupic H, Kasuba V, Mikecin AM, Grdisa M. Tissue extract from Eisenia foetida as a wound-healing agent. Eur Rev Med Pharmaco 2010; 14(3):177-184

20. Li DM, Liu DS**.** Observation on the effect of Dilong ointment on postoperative wound healing of anorectal disease. New progress in research on anorectal diseases of integrated Chinese and Western medicine. Shenyang; 2000:522-523. (in Chinese)

21. Duan XJ, Luo SL, Wang XF, Yang YW, Geng D, Zhao RY, Zheng ZH, Ding YT, Sun YK. Active fraction of DL3 and its would healing study. Information on Traditional Chinese Medicine 2018; 35(1):9-11(in Chinese)
