# Supplementary material for: Effect of Earthworm on Wound Healing: A Systematic Review and Meta-Analysis
Source: Front Pharmacol. 2021 Oct 22;12:691742. doi: 10.3389/fphar.2021.691742 (PMC8568887; doi:10.3389/fphar.2021.691742)

**PubMed:**

((((((((((wound healing[Title/Abstract]) OR (wound healings[Title/Abstract])) OR (healing, wound[Title/Abstract])) OR (healings, wound[Title/Abstract])) OR (regeneration[Title/Abstract])) OR (injury repair[Title/Abstract])) OR (wound repair[Title/Abstract])) OR (re epithelialization[Title/Abstract])) OR (wound epithelialization[Title/Abstract])) OR ("Wound Healing"[Mesh])) AND ((((((((((((((((((((Oligochaeta[Title/Abstract]) OR (Oligochaetas[Title/Abstract])) OR (Dilong[Title/Abstract])) OR (Di Long[Title/Abstract])) OR (Earthworms[Title/Abstract])) OR (Earthworm[Title/Abstract])) OR (Lumbricus terrestris[Title/Abstract])) OR (Lumbricus terrestri[Title/Abstract])) OR (terrestris, Lumbricus[Title/Abstract])) OR (Lumbricus[Title/Abstract])) OR (Eisenia worm[Title/Abstract])) OR (Eisenia worms[Title/Abstract])) OR (worm, Eisenia[Title/Abstract])) OR (Eisenia foetida[Title/Abstract])) OR (Eisenia foetidas[Title/Abstract])) OR (foetidas, Eisenia[Title/Abstract])) OR (Eisenia fetida[Title/Abstract])) OR (Eisenia fetidas[Title/Abstract])) OR (fetida, Eisenia[Title/Abstract])) OR ("Oligochaeta"[Mesh]))

**EMBASE:**


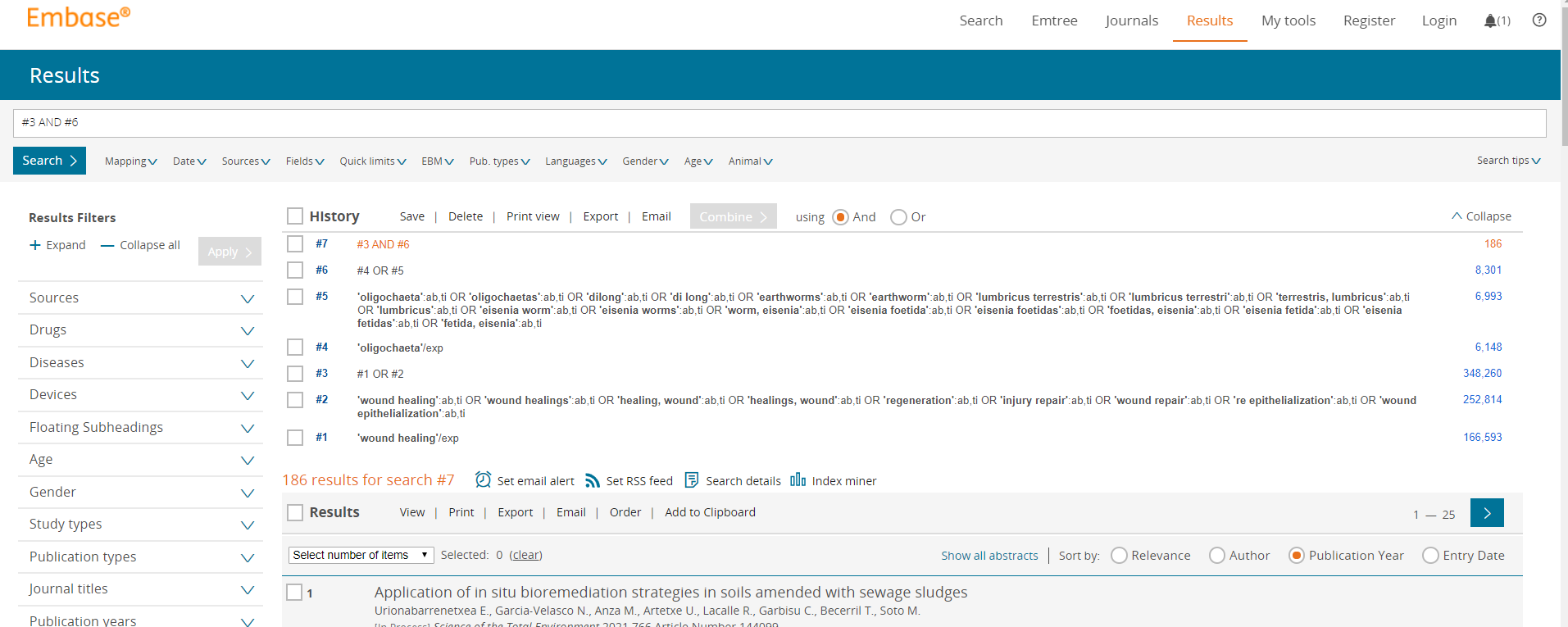


**Web of Science:**


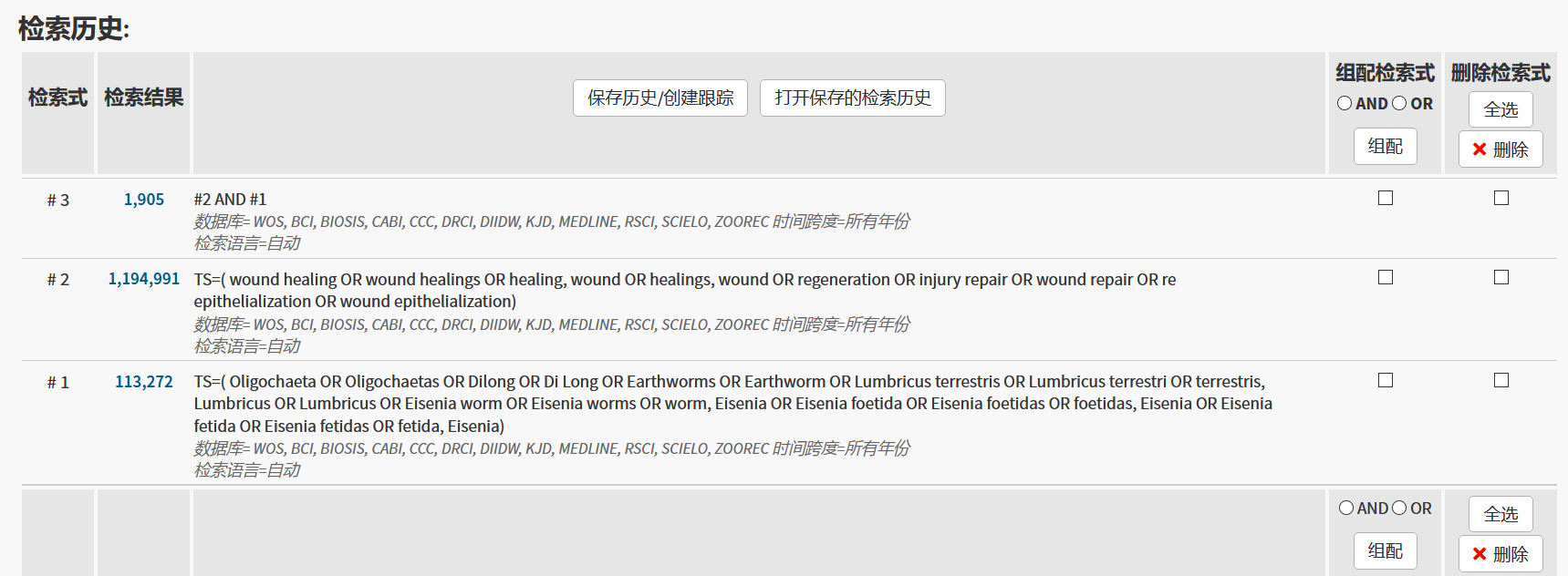


**the Cochrane Library:**


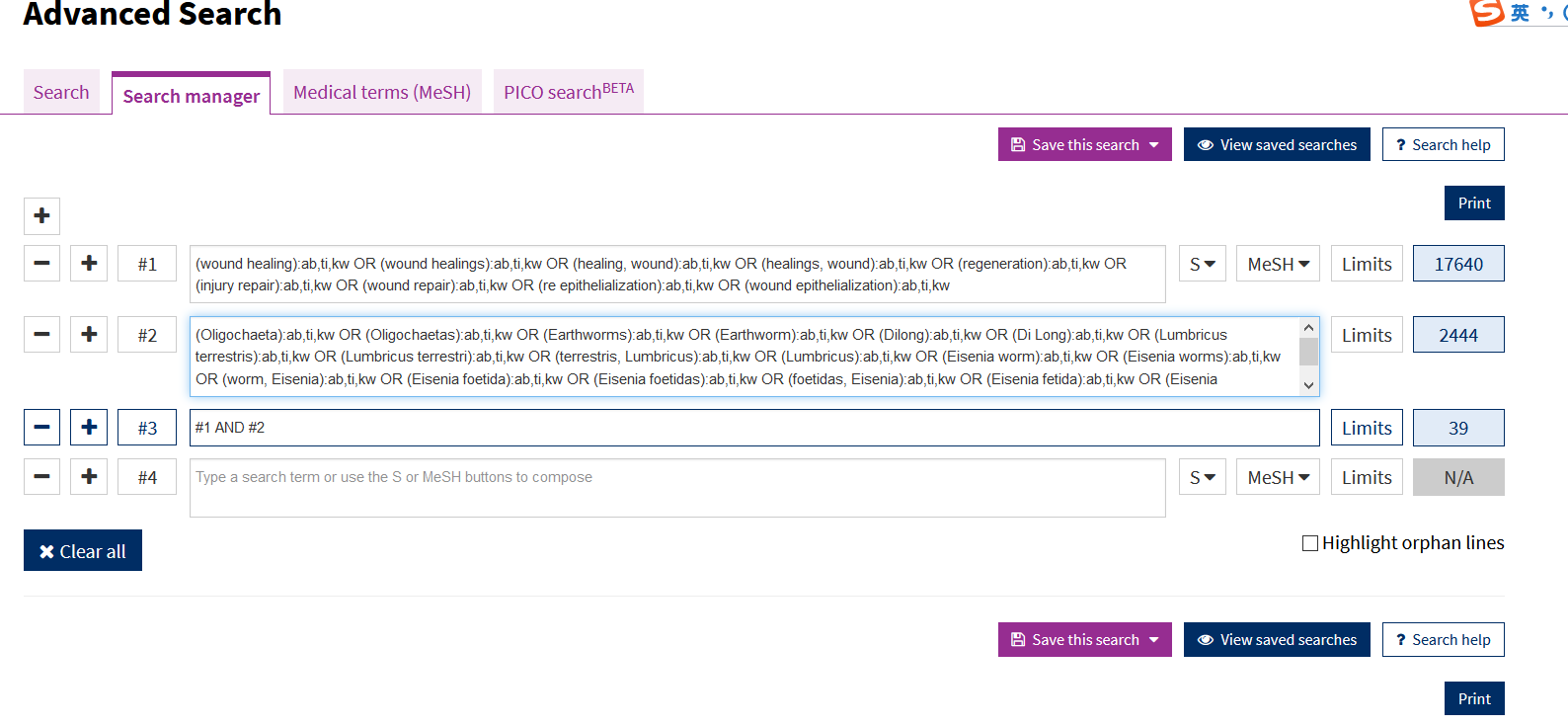


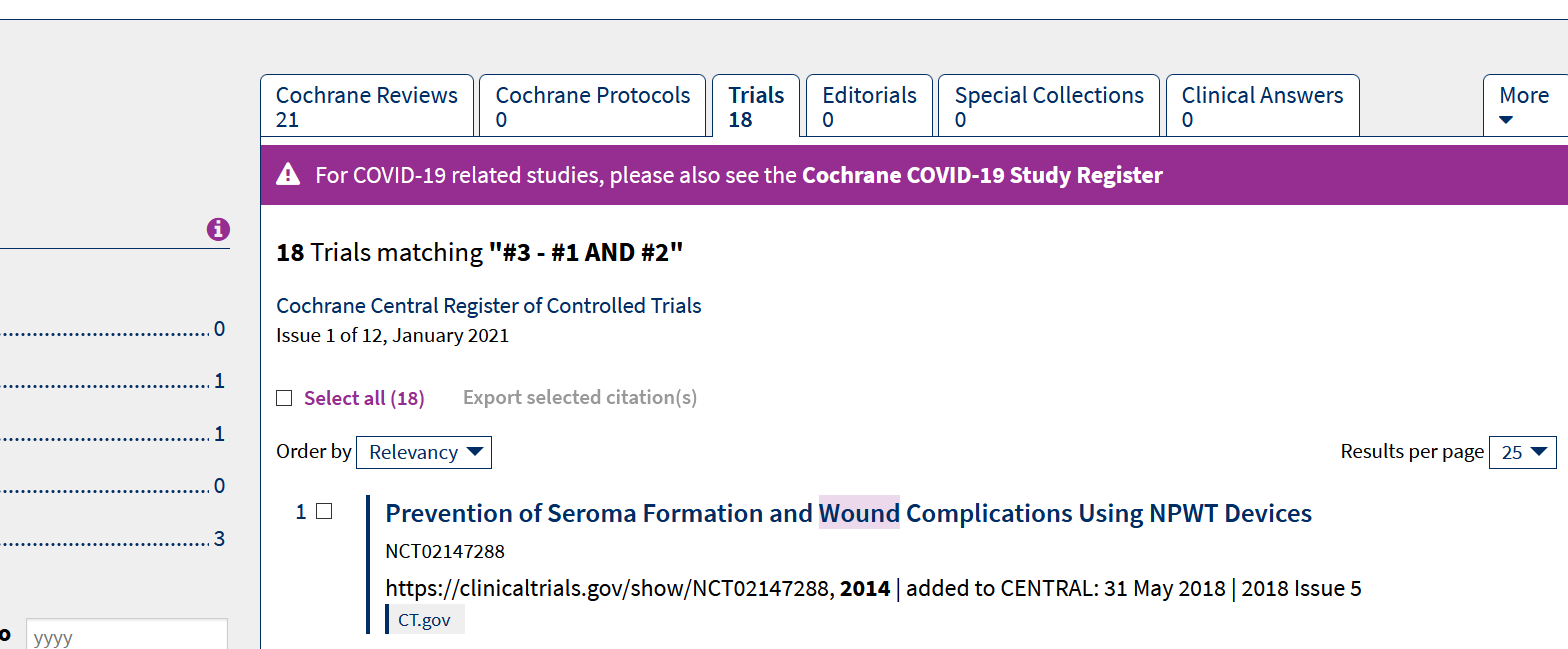


**CNKI:**


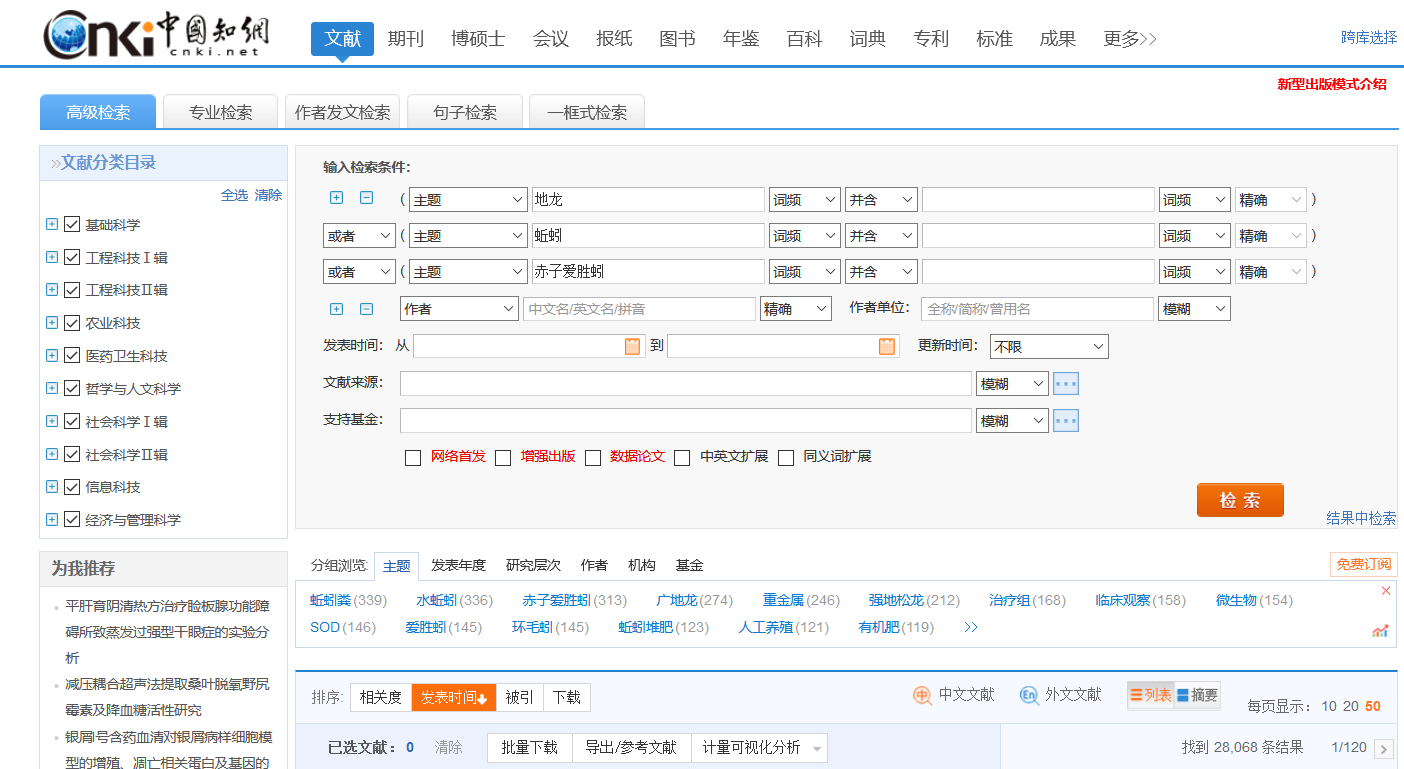


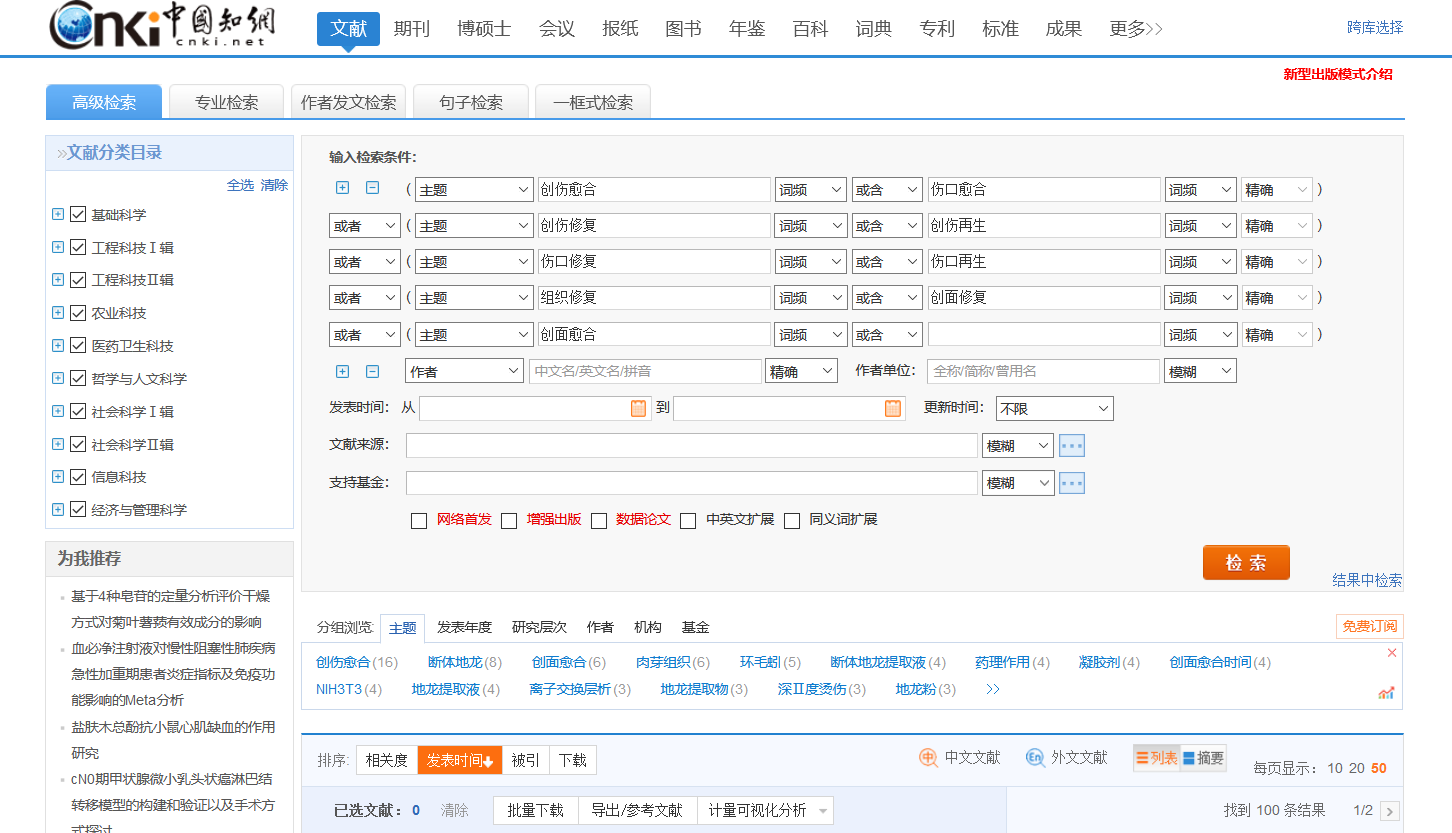


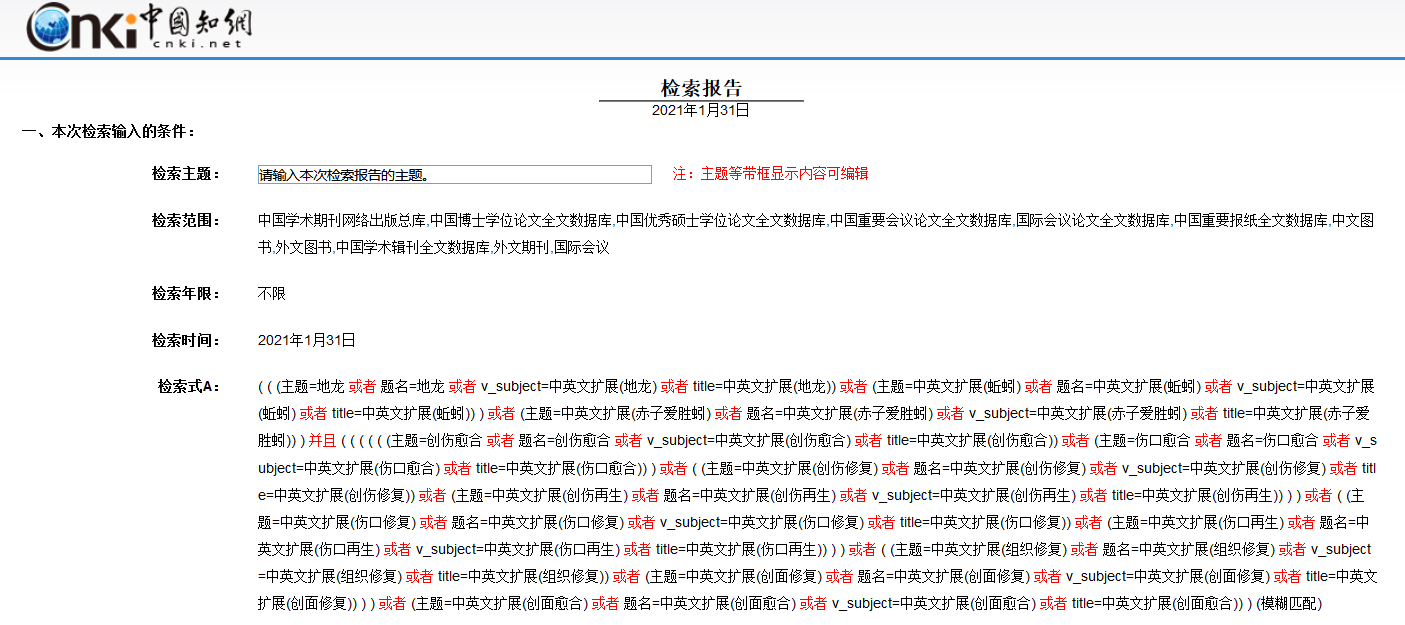


**VIP:**


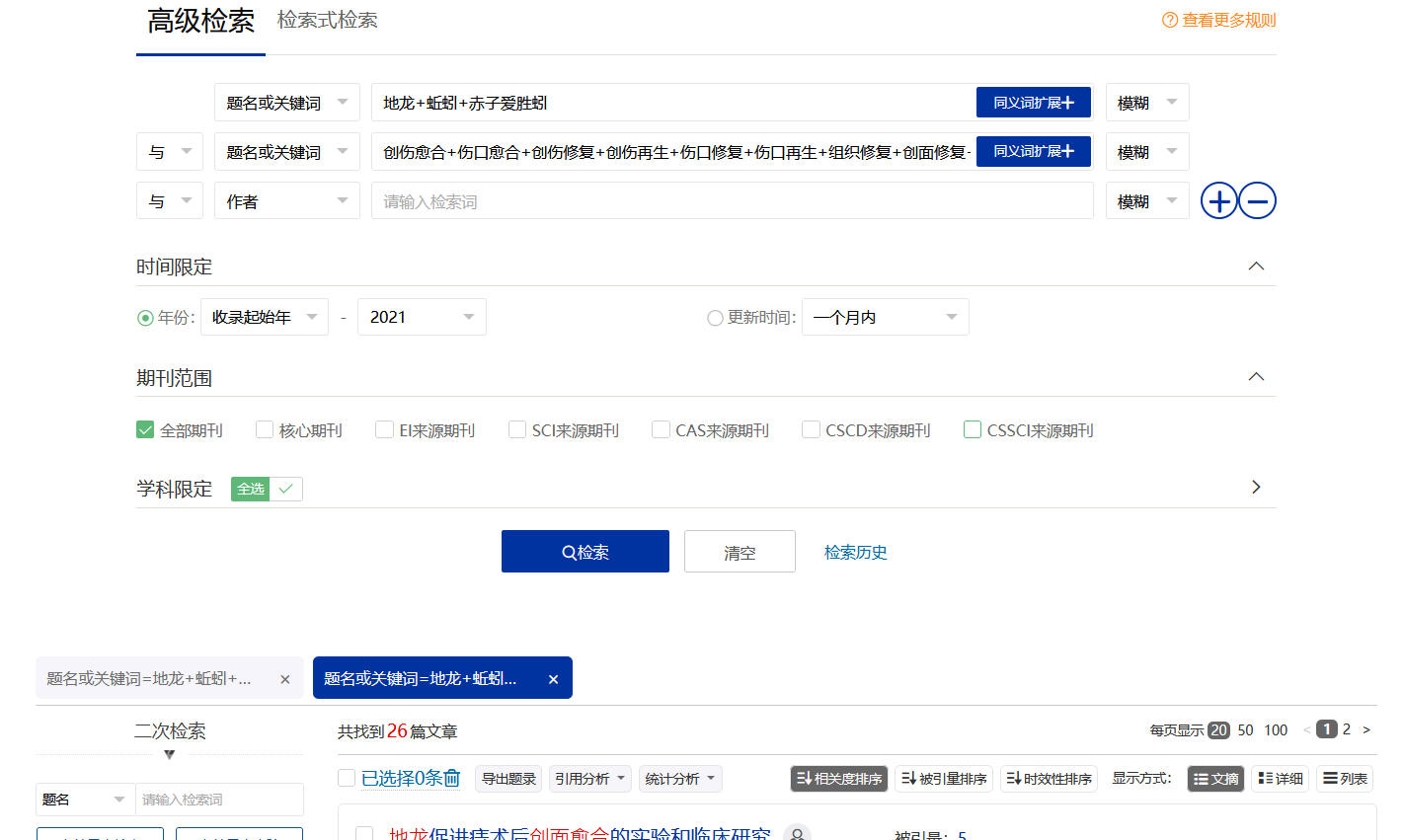


**WanFang:**


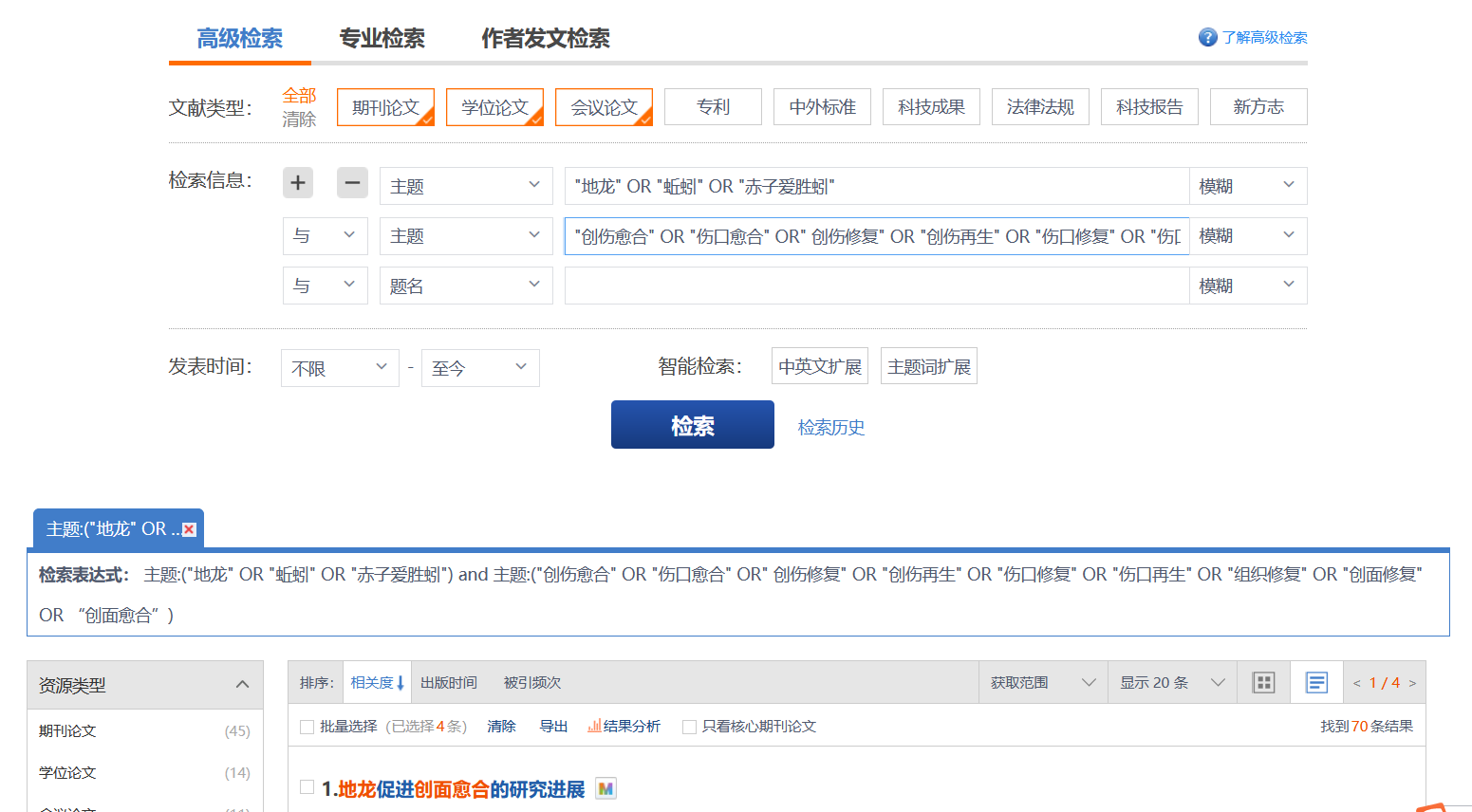

Supplement: Supplementary file 5 [file DataSheet1.DOCX]
